# Supplementary material for: Low-frequency vibrational density of states of ordinary and ultra-stable glasses
Source: Nat Commun. 2024 Feb 16;15:1424. doi: 10.1038/s41467-024-45671-8 (PMC11258317; doi:10.1038/s41467-024-45671-8)
Supplement: Supplementary file 1 — Supplementary Information [file 41467_2024_45671_MOESM1_ESM.pdf]

Supplementary information for "Low-frequency vibrational density of states of ordinary and ultra-stable glasses"

Ding Xu[†], Shiyun Zhang[†], Hua Tong, Lijin Wang[*], Ning Xu[*]

Corresponding author: lijin.wang@ahu.edu.cn, ningxu@ustc.edu.cn

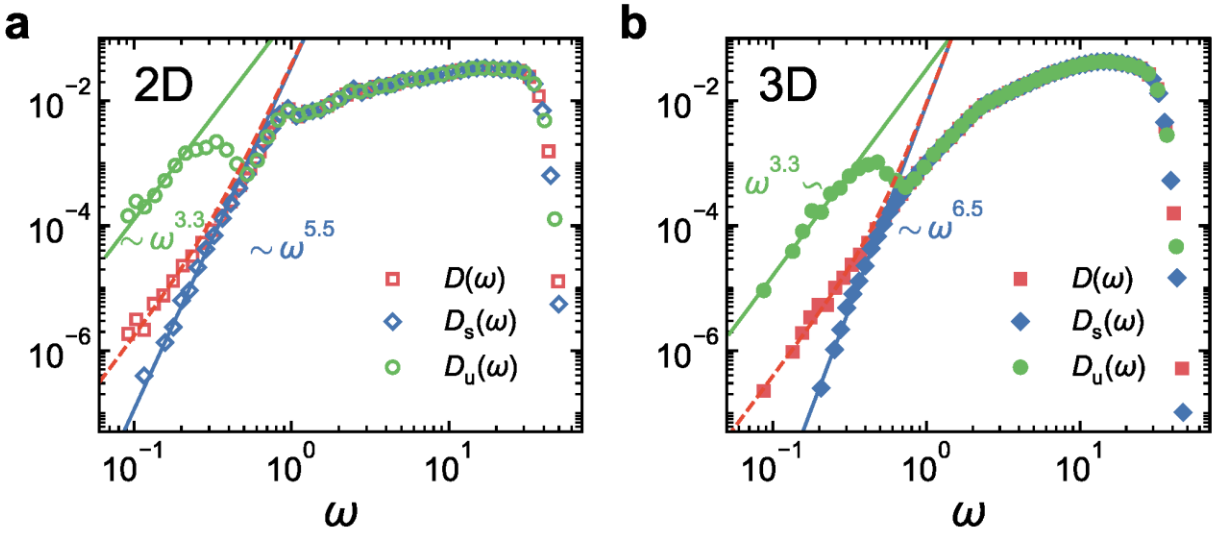

Supplementary Figure 1. Comparison of VDOS's of stable, unstable, and all glasses with the Lennard-Jones interaction. a Results of a 2D system with $N = 1000$. **b** Results of a 3D system with $N = 1000$. The solid lines are power-law fittings to $D_s(\omega)$ and $D_u(\omega)$ at low frequencies. The red dashed lines are results from Eq. (1) of the main text. The systems studied here are the Kob-Andersen Lennard-Jones binary mixtures with the number density $\rho = 1.2$ in both 2D and 3D. To suppress crystallization, the number ratio of the two types of particles used here is 65:35 (80:20) in 2D (3D). We can see exactly the same results as the IPL systems shown in the main text, including the values of the scaling exponents.

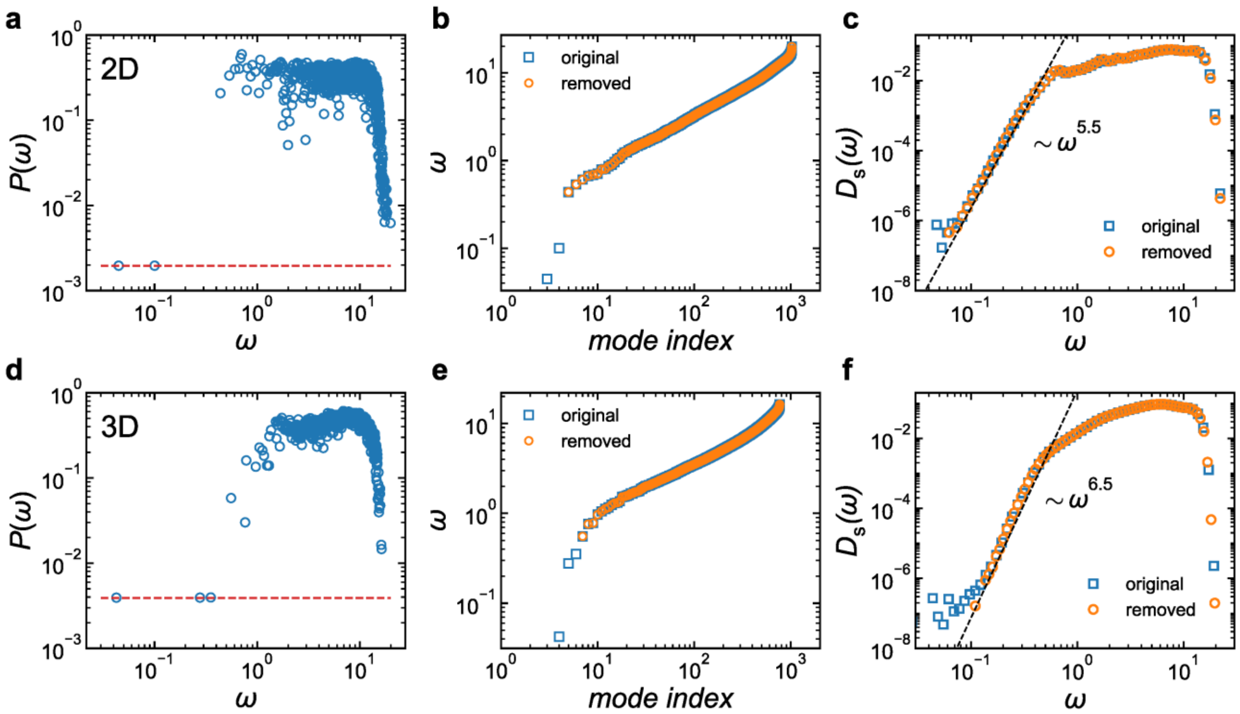

Supplementary Figure 2. Localized modes caused by rattler-like particles. a-c are results of 2D solids consisting of $N = 512$ particles interacting via the IPL potential and quenched from a parent temperature $T_{\rm p} = 0.25$. **d-f** are results of 3D solids consisting of $N = 256$ particles interacting via the IPL potential and quenched from a parent temperature $T_{\rm p} = 0.18$. **a,d** Example of the participation ratio $P(\omega)$ of all normal modes of vibration for a special stable solid with localized low-frequency modes. The horizontal dashed lines locate $P(\omega) = 1/N$. A mode with such a participation ratio is localized, i.e., only one particle effectively vibrates in the mode. For a quite small fraction of stable solids, we can see d such localized modes (d is the spatial dimension), suggesting that there is a rattler-like particle. **b,e** Frequencies of all modes before and after the rattler-like particle is removed. The d lowest frequencies of the "original" data are associated with the rattler-like particle. After the particle is removed, the frequencies of the "removed" data collapse with the "original" data, verifying that the particle is indeed rattler-like. **c,f** VDOS of stable solids before and after the rattler-like particles are removed. The rattler-like particles contribute a low-frequency tail in the "original" VDOS. Because these particles are effectively rattlers, we only show the VDOS after they are removed in the main text. Note that the rattler-like particles should only exist in systems with short-range interactions. Whether the low-frequency tail caused by the rattler-like particles needs to be seriously taken into account requires further investigations.
